# Pipeline flow diverter and transvenous coiling for the treatment of direct carotid cavernous sinus fistulae: a retrospective case series

**DOI:** 10.1186/s42155-025-00566-7

**Published:** 2025-05-17

**Authors:** Omar Abu-Fares, Antonis Adamou, Heinrich Lanfermann, Joachim K. Krauss, Shadi Al-Afif, Katja Döring

**Affiliations:** 1 Department of Diagnostic and Interventional Neuroradiology, Hannover Medical School Hannover, 30625 Hannover, Germany; 2 Department of Neurosurgery, Hannover Medical School, 30625 Hannover, Germany

**Keywords:** Dural arteriovenous fistula, Pipeline Vantage flow diverter, Anatomy

## Abstract

**Background:**

Carotid cavernous fistulae (CCF) are pathological connections between the carotid arteries and the cavernous sinus. Endovascular procedures are the mainstay treatment for CCF. The aim of this report is to evaluate the efficacy and safety of the combined use of the Pipeline Vantage (PV) flow diverter and transvenous coil embolization in the treatment of CCF.

**Methods:**

Retrospective,monocentric analysis of three patients who underwent neurointerventional treatment of clinically symptomatic CCF using a combination of transvenous coil embolization and flow diverter Implantation. Clinical data, the etiology of the CCF and clinical and radiological follow up were evaluated.

**Results:**

Clinical and radiological follow-up were available at 3 and 12 months. One patient experienced clinical improvement immediatley after the interventions. The other two patients improved within one year after treatment. No symptomatic complications were recorded. One year after the intervention complete CCF occulsion was documented in all cases.

**Conclusions:**

We report an initial experience for the treatment of direct CCF using the combination of a new generation Pipeline FD and transvenous coil embolization. The high treatment success rate and low complication rate are encouraging.

## Introduction

Carotid cavernous sinus fistula (CCFs) are pathological connections between the carotid arteries and the cavernous sinus. They are usually classified as direct or indirect, high or low flow, or according to the Barrow classification [1]. Barrow type A CCF is a direct high-flow connection between the internal carotid artery (ICA) and the cavernous sinus. Type B, C and D arteriovenous fistulae are called"indirect"because they connect between branches of the ICA and/or the external carotid artery (ECA) with the cavernous sinus and are usually low flow [1, 2].

The main symptoms of CCF are double vision due to pressure damage to the cranial nerves responsible for eye movement (cranial nerves III, IV and VI) in the cavernous sinus and a pulsatile tinnitus. Proptosis, chemosis, decreased visual acuity, diplopia, and eye pain due to obstruction of the outflow of the superior ophthalmic vein caused by the arterial inflow from the carotid artery are also common. The choice of treatment depends mainly on the type of the arteriovenous fistula as well as the patient’s symptoms. Treatment should be offered for all high-flow or Barrow type A fistulae, as the likelihood of the patient's symptom worsening is high and intracranial hemorrhage due to cerebral venous congestion is a risk. Stent- or balloon-assisted coil-embolization of the cavernous sinus is considered the first-line treatment for direct CCFs.

Flow diverter (FD) has become an important tool for the treatment of intracranial aneurysms. FD stents differ from conventional intracranial stents in its finely woven mesh with greater vessel wall metal coverage, higher pore density and lower porosity. When implanted in the parent vessel, the device diverts the flow away from the aneurysm, causing secondary blood stasis and subsequent thrombosis of the aneurysm. After a few months, neoendothelium forms over the mesh, reconstructing the parent vessel and completely occluding the aneurysm neck [3, 4]. In recent years, there is a growing trend towards off-label use of these devices [5]. The aim of this study is to give an overview for the treatment of CCF and our experience in using a new generation pipeline FD with adjunctive transvenous coiling in the treatment of direct CCF.

## Material and methods

### Patient selection

Patients undergoing treatment for a direct CCF using Pipeline Vantage (PV) FD and transvenous coiling were included in this retrospective, single-center study. Written informed consent was obtained for the endovascular procedure. For each patient, medical history, neurologic status, demographic data as well as clinical and radiological outcomes were evaluated.

## Results

Three female patients (51—70 years) were identified. All patients underwent transarterial FD Implantation and transvenous coil-embolization. In total 5 FDs were implanted. Patient and CCF characteristics are summarized in Table 1.

**Table 1:** Baseline data

No.	Age [years]	gender	Etiology	Number of flow diverters/Size	Adjunctive venous coiling	Fistula occlusion	Increased intraocular pressure and/or decreased visual acuity
1	70	female	iatrogenic	1/4.5x25 mm	yes	immediate	yes
2	69	female	idiopathic	3/4.5x16 mm	yes	on follow up imaging	no
				5.5x16 mm			
				6x30 mm			
3	51	female	traumatic	1/5x30 mm	yes	on follow up imaging	no

### Case illustrations

#### Case I

**Fig. 1:**
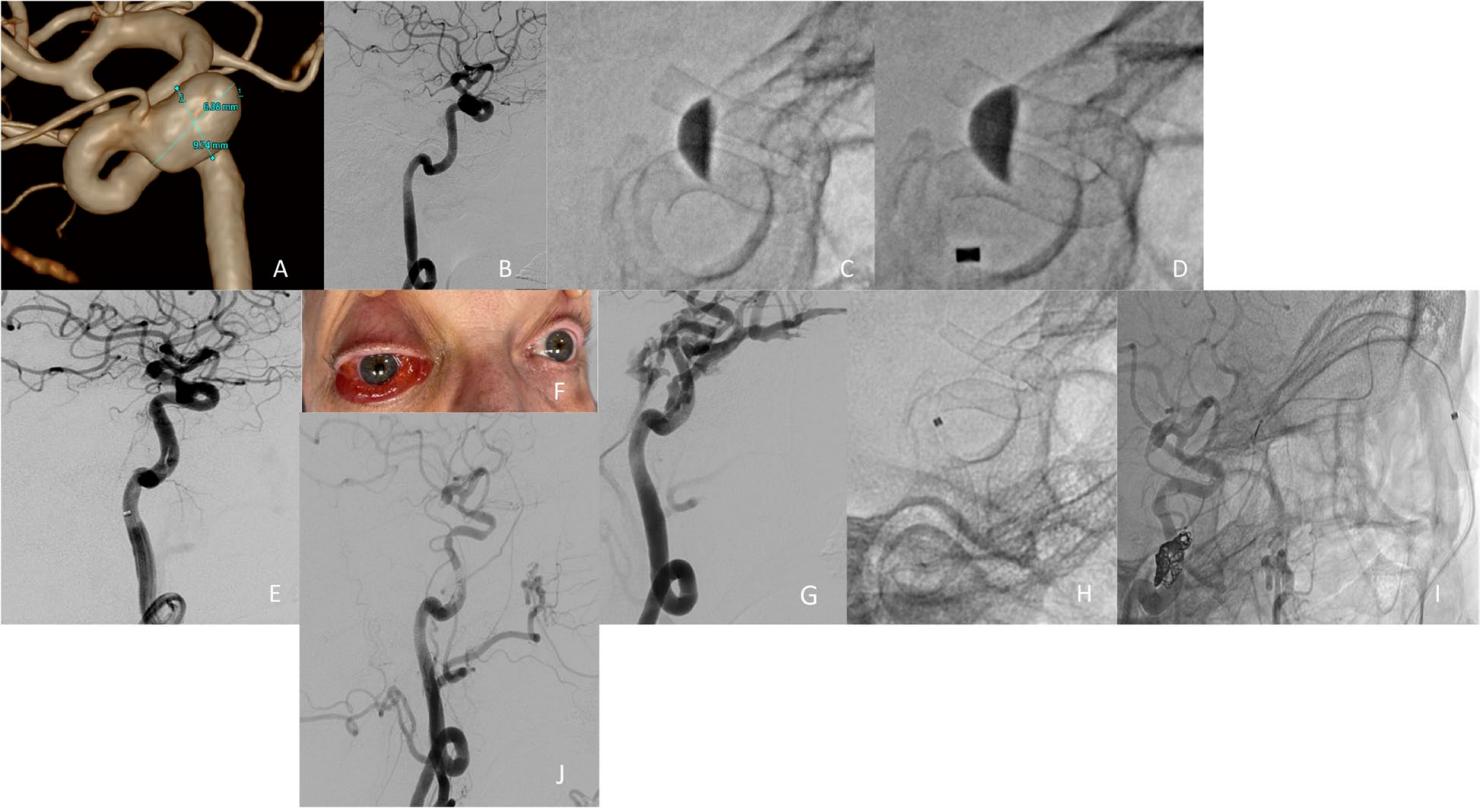
Case I

In December 2022, a 70-year-old woman underwent a diagnostic MRI to investigate vestibular loss. An incidental right ophthalmic ICA aneurysm of 6 × 9 mm was detected. Six months later, the aneurysm was confirmed by digital subtraction angiography (DSA) (Fig. 1A, 1B) and the patient underwent aneurysm treatment using the Pipeline Vantage (PV) FD. FD twisting in the ophthalmic ICA genu was adjusted using balloon remodeling (Fig. 1C, 1D). In the final DSA run after FD deployment, a dissection of the ICA cavernous segment was observed (Fig. 1E). However, since the dissection did not compromise blood flow in the ICA conservative therapy was determined. In November 2023, the patient presented to the Emergency Department with proptosis, chemosis, tinnitus and decreased visual acuity of the right eye (Fig. 1F). The subsequent DSA documented a direct CCF at the level of the ICA dissection with venous drainage to the superior and inferior ophthalmic veins and the ipsilateral inferior petrosal sinus (Fig. 1G). Treatment of the CCF was performed by implanting FD in the right ICA cavernous segment as well as transvenous coiling of the cavernous sinus.

#### Endovascular procedure

A long 6 F neuron max sheath was introduced through the left femoral artery to the right ICA. Due to ICA elongation, a distal access catheter (DAC) Navien 5f was introduced to the distal cervical ICA. Access to the ICA distal to the dissection was achieved using Synchro 14 microwire and Phenom 27 Microcatheter. A PV FD 4.5 × 25 mm was then deployed over the dissection (Fig. 1H). After FD Implantation, a right femoral venous access was obtained. Then a 6 F Envoy catheter was introduced to the right internal jugular vein. A DAC Phenom plus catheter with Excelsior SL 10 microcatheter and Synchro 14 microwire were then introduced to the facial vein and subsequently to the ophthalmic vein (F ig. 1I). The cavernous sinus at the level of the fistula was then occluded with 3 Target Coils. On the final DSA run, the fistula was cured (Fig. 1J).

#### Radiographic and clinical follow up

One day after the procedure there was a rapid improvement of the clinical symptoms on the right eye and the tinnitus disappeared. Clinical and MRI follow up 3 months after the procedure confirmed complete recovery of the symptoms and no signs of fistula-recurrence. Dual antiplatelet therapy (DAPT) was modified to Acetylsalicylic acid (ASA) 100 mg daily. Clinical follow up one year after the procedure was uneventful.

#### Case II

**Fig. 2:**
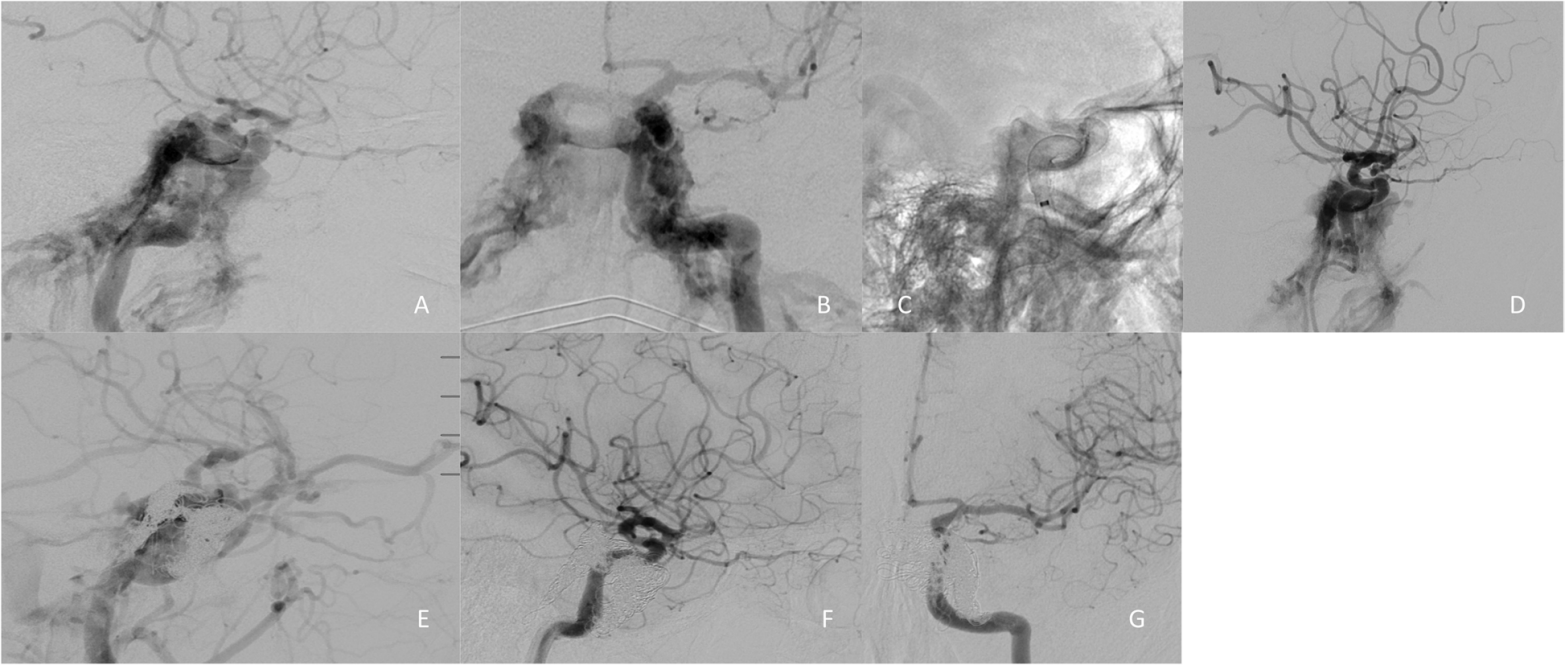
Case II

In January 2023, a 69-year-old woman presented in the emergency department with a pulse-synchronous tinnitus in the left ear and left-sided neck pain. MRI exam performed as part of the diagnostic work-up showed increased flow voids in the cavernous sinus and middle cranial fossa along the bony borders of the left sphenoidal sinus and lateral orbital funnel. DSA confirmed the diagnosis of a direct left CCF (Barrow A type) with no evidence of cerebral or orbital congestion (Fig. 2A and 2B). In addition, a left paraclinoidal ICA aneurysm of 3 mm was documented. A decision was made to treat the fistula as well as the left ICA aneurysm with flow diverter and adjunctive transvenous coil embolization.

#### Endovascular procedure

A 6f long neuron max sheath was introduced through the right femoral artery and advanced to the left ICA. A DAC Navien with Phenom 27 microcatheter and Synchro m14 microwire were then advanced to the distal ICA. Three PV FDs (4.5 × 16 mm, 5.5 × 16 mm and 6 × 30 mm) were placed from the ophthalmic to petrous ICA segments (Fig. 2C). Final DSA Run documented persistent high-flow direct CCF (Fig. 2D).

Five Weeks later, a second intervention was performed. A Neuron max long sheath was introduced via right femoral vein into left internal jugular vein. A DAC 5f Sofia with Excelsior SL 10 microcatheter and synchro 14 microwire were then advanced in to the inferior petrosal sinus and then in to the cavernous sinus. Coilembolization of the left cavernous sinus und intercavernous sinus was performed. On the final DSA run the high flow fistula was still observed with orbital venous congestion (Fig. 2E).

#### Radiographic and clinical follow up

After the intervention intraocular pressure was normal, so further embolization was not carried out.

Postinterventional clinical and radiological follow up was available at 3 months and at one year. No complication were documented. DAPT therapy was modified three months after the intervention to 100 mg ASA daily. After discontinuation of the DAP medication rapid improvement of tinnitus occurred. DSA performed one year after the intervention documented complete occlusion of the fistula and patent left ICA (Fig. 2F and 2G).

#### Case III

**Fig. 3:**
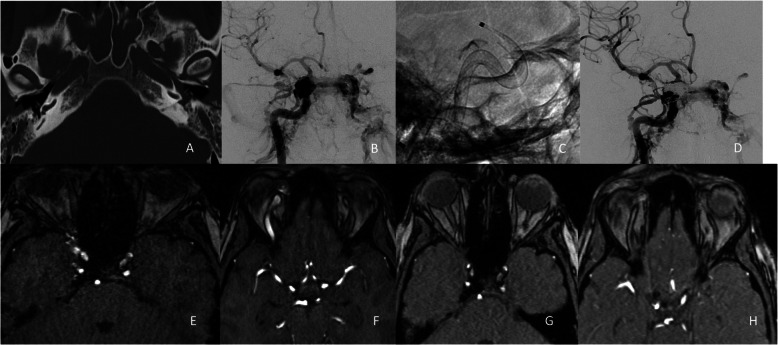
Case III

In June 2023, a 51-year-old woman suffered a road traffic accident while riding her e-bike. She sustained a complex skull base fracture involving the bilateral petrous temporal bones and carotid canal (Fig. 3A). The acceleration trauma resulted in a dissecting aneurysm of the left ICA in pars lacerum (C3 segment) and a traumatic direct carotid cavernous sinus fistula on the right, with venous drainage mainly to the cavernous sinus and right superior petrosal sinus (Fig. 3B). The decision was made to treat the fistula and the aneurysm with FDs. In addition venous coil embolization of the right cavernous sinus was carried out.

#### Endovascular procedure

Through a right femoral access a 6 F Neuron max sheath was introduced to the right ICA. A DAC Navien 5 F with Phenom 27 microcatheter and Synchro 14 microwire were then advanced to the M1 Segment of the middle cerebral artery (MCA). Then, a PVFD 5 × 30 mm was deployed in the ICA covering the ophthalmic and cavernous ICA segments (Fig. 3C). Following that, a 6 F Envoy catheter was advanced through a left femoral sheath to the left internal jugular vein. Excelsior SL 10 microcatheter and a Synchro 14 microwire were then advanced through the superior petrosal sinus to the right cavernous sinus. 10 Target Coils were then deployed in the right cavernous sinus. In the final DSA run reduced volume shunting through the fistula was observed and ICA opacification was enhanced (Fig. 3D).

#### Radiographic and clinical follow up

Postinterventionally, no symptomatic complications were documented. On control MRI performed after the intervention, persistent flow voids in the right cavernous sinus and dilated ophthalmic vein on the right side were still observed (Fig. 3E and 3 F). Three months following the procedure DAPT was discontinued and the MRI follow up documented resolution of the fistula (Fig. 3G and 3H). Patient’s symptom of tinnitus resolved. One year after the procedure no adverse events were reported and follow up MRI did not show any signs of fistula-recurrence.

#### Dual antiplatelet therapy

All patients received a loading dose of 100 mg ASA and 600 mg clopidogrel the night before the procedure. On the morning of the procedure, a repeat loading dose of 100 mg ASA and 300 mg clopidogrel was administered. Starting the day after the procedure, patients received a maintenance regimen of 100 mg ASA and 75 mg clopidogrel for 3 months, followed by indefinite monotherapy with 100 mg ASA once daily. Platelet function tests were not performed.

### Safety and efficacy

In all three cases, FD implantation was uneventful. Successful FD deployment was achieved in all cases and no periprocedural complications were documented. Clinical and radiological follow up for all patients was available at 3 and 12 months. Complete CCF treatment was documented on follow up imaging and there were no signs of fistula- recurrence. Ocular symptoms and tinnitus remained resolved in all patients.

## Discussion

CCF is a rare disease with an overall incidence rate of 0.37 per 100,000 per year [6]. Clinical signs and symptoms associated with a poor prognosis include increased intracranial pressure, rapidly progressive proptosis, decreased visual acuity, cerebral hemorrhage and transient ischemic attacks. The latter are often caused by impaired autoregulation of cerebral perfusion as a result of the chronic steal phenomenon caused by the fistulous connection [7, 8]. Halbach et al. collected angiographic and clinical data from approximately 155 CCF patients to identify features that warrant immediate and aggressive interventional treatment [9]. Angiographic features include the presence of a pseudoaneurysm, a large cavernous sinus varix, venous drainage into the cortical veins, and thrombosis of other venous outflow pathways distant from the fistula. In particular, cortical venous congestion carries a risk of hemorrhage and should therefore be treated [10].

There are different treatment options for CCF; conservative treatment, which mainly consists of manual compression, or endovascular intervention (transarterial or transvenous), which is the mainstay treatment approach. Although the clinical manifestations of direct and indirect fistulae may overlap, the endovascular treatment methods are quite different. The choice of treatment depends on the type and anatomy of the fistula and the size of the arterial defect. While direct fistulae are caused by a rupture in the cavernous segment of the ICA or, less commonly, by the intracavernous rupture of an ICA aneurysm, indirect fistulae are small dural arteriovenous shunts between the meningeal branches of the ICA, ECA (or both) and the cavernous sinus. Accordingly, the aim of treatment of direct CCFs is to close the fistula between the ICA and the cavernous sinus while maintaining patency of the ICA. For indirect fistulae, the aim is to interrupt the fistulous connections and reduce the pressure in the cavernous sinus. The following sections provide a brief overview of the endovascular treatment options of direct and indirect CCFs.

### Endovascular therapy: direct CCFs

Serbinenko et al. published the first case of successful endovascular embolization of a CCF using a removable silicone balloon with preservation of the ICA [11] However, If the fistula is too large, the embolization balloon can shift back into the ICA when inflated in the cavernous sinus [12]. Typically, inadequate embolization can occur due to premature detachment of the balloon, deflation or rupture due to contact with a bone fragment [13]. Due to the limited availability of detachable balloons, transarterial embolization with coils or other embolic material is now the mainstay of endovascular treatment of direct high-flow CCFs [14]. Embolization can be performed with coils and liquid embolic agents [14]. In the standard transarterial approach, a guide catheter is inserted into the cervical ICA. A microcatheter is then advanced superselectively into the cavernous segment through the tear in the ICA. The embolic material is then delivered into the cavernous sinus via the microcatheter [14]. Detachable coils are preferred due to their reliable and controlled placement. Common complications of transarterial coil embolization include thromboembolism, impaired ICA flow dynamics due to protruding coil mass and ICA dissection [7]. To prevent retrograde herniation of embolic material into the parent artery and distal intracranial circulation, support with a non-detachable balloon (balloon-assisted) or a porous stent (stent-assisted) may be considered [7, 14].

### Endovascular therapy: Indirect CCFs

Transvenous embolization is the preferred treatment approach for indirect CCFs. The aim is to catheterize the cavernous sinus superselectively and close the fistula without diverting venous outflow to cortical structures [7, 14]. Different embolic materials such as coils and liquid embolic can be used alone or in combination. The advantages of coils include their radiopacity, ease of application and the ability to reposition or remove them if the original placement proves to be suboptimal. Limitations include the difficulty of achieving complete occlusion with adequate volumetric packing as well as increased risk of cranial nerve palsy [14]. Therefore, transvenous liquid embolic agents are increasingly being used, either alone or in combination with platinum coils. Unfortunately, liquid embolic agents have a tendency to retrograde fill the arterial supply and must therefore be used with caution [14].

### Flow Diversion

FD stents have much denser mesh than conventional stents and act as a scaffold by reconstructing the original vessel wall due to neointima building. Originally, FDs were designed to treat complex aneurysms, such as broad-based, fusiform or giant aneurysms. None of the commercially available FDs are formally approved by the FDA for the treatment of CCF. However, some have been successfully used off-label either as sole or in combination with coils for the treatment of direct CCF. The advantages of the FD are based on two effects: first, FD forms a scaffold which ensures protection of the parent vessel during transvenous embolization. Due to the smaller mesh size of the FD, coil protrusion into the parent vessel is less likely when coil embolization is combined with FD compared to other stents, and subsequently may reduce thromboembolic complications. In addition, the flow diversion effect in addition to neointima formation induced by the FD favors thrombosis of incompletely occluded fistulae. The disadvantages of this method are manageable. As all braided stents, FDs could be difficult to deploy in tortuous vessels. More importantly, DAPT is required for a period of time after FD implantation and prolonged obliteration of the fistula is to be expected.

Fourth generation PVFD was used in our study. This device has several modifications over its predecessor designed to improve insertion, visibility, distal opening as well as deployment. Compared to the Pipeline Shield FD, the wires of the PVFD have a smaller diameter and the device has a thinner wall [15]. This characteristic has the potential to promote endothelial growth over the struts of the FD and thus improve the flow diversion effct.

Initial endovascular treatment attempts of CCF using FDs have been made. In 2017, Wendl et al. published a collective of 14 patients treated with FD for direct CCF between 2011 and 2015 [16]. Overall 59 FDs were implanted. 10/14 patients were free from ocular symptoms (71%), 2 had residual proptosis, and no patient had clinical deterioration at the last follow up. They also reported two asymptomatic occlusions of the ICA that were related to an interruption of antiaggregation. Despite the use of many FDs, complete occlusion of the ICA only occurred weeks to months later, making long-term and continuous anticoagulation necessary.

## Conclusion

Our report provides an alternative therapeutic strategy for the treatment of direct CCF utilizing a new generation PV FD in combination with transvenous coil embolization. The high treatment success rate and low complication rate are encouraging. Further studies with larger cohorts are needed to further validate safety and efficacy.

## Data Availability

Not applicable.

## Abbreviations

ASA: Acetylsalicylic acid
CCF: Carotid cavernous sinus fistula
CT: Computer tomography
DAC: Distal access catheter
DAPT: Dual antiplatelet therapy
DSA: Digital subtraction angiography
ECA: External carotid artery
FDA: U.S. Food and Drug Administration
FD: Flow Diverter
ICA: Internal carotid artery
MCA: Middle cerebral artery
MRI: Magnetic resonance imaging
PV: Pipeline Vantage
ToF: Time of flight

## References

[1] Barrow DL, Spector RH, Braun IF, Landman JA, Tindall SC, Tindall GT. Classification and treatment of spontaneous carotid-cavernous fistulas. J Neurosurg. 1985;62(2):248–56. 3968564 10.3171/jns.1985.62.2.0248

[2] Kashiwagi S, Tsuchida E, Goto K, Shiroyama Y, Yamashita T, Takahasi M, et al. Balloon occlusion of a spontaneous carotid-cavernous fistula in Ehlers-Danlos syndrome type IV. Surg Neurol. 1993;39(3):187–90. 8456380 10.1016/0090-3019(93)90180-9

[3] Kallmes DF, Ding YH, Dai D, Kadirvel R, Lewis DA, Cloft HJ. A new endoluminal, flow-disrupting device for treatment of saccular aneurysms. Stroke. 2007;38(8):2346–52. 17615366 10.1161/STROKEAHA.106.479576

[4] Ravindran K, Salem MM, Alturki AY, Thomas AJ, Ogilvy CS, Moore JM. Endothelialization following flow diversion for intracranial aneurysms: a systematic review. AJNR Am J Neuroradiol. 2019;40(2):295–301. 30679207 10.3174/ajnr.A5955 PMC7028638

[5] Ogilvy CS, Motiei-Langroudi R, Ghorbani M, Griessenauer CJ, Alturki AY, Thomas AJ. Flow Diverters as Useful Adjunct to Traditional Endovascular Techniques in Treatment of Direct Carotid-Cavernous Fistulas. World Neurosurgery. 2017;105:812–7. 28647659 10.1016/j.wneu.2017.06.113

[6] Cohen DA, Sanchez Moreno FR, Bhatti MT, Lanzino G, Chen JJ. Evaluating the Incidence and Neuro-Ophthalmic Manifestations of Carotid-Cavernous Fistulas. J Neuroophthalmol. 2024;44(2):232–5. 10.1097/WNO.0000000000001973. Epub 2023 Aug 14. PMID: 37581565; PMCID: PMC10864674. 37581565 10.1097/WNO.0000000000001973 PMC10864674

[7] Ringer AJ, Salud L, Tomsick TA. Carotid cavernous fistulas: anatomy, classification, and treatment. Neurosurg Clin N Am. 2005;16:279–95. 15694161 10.1016/j.nec.2004.08.004

[8] Meyers PM, Halbach VV, Dowd CF, Lempert TE, Malek AM, Phatouros CC, Lefler JE, Higashida RT. Dural carotid cavernous fistula: definitive endovascular management and long-term follow-up. Am J Ophthalmol. 2002;134:85–92. 12095813 10.1016/s0002-9394(02)01515-5

[9] Halbach VV, Hieshima GB, Higashida RT, Reicher M. Carotid cavernous fistulae: indications for urgent treatment. AJR Am J Roentgenol. 1987;149:587–93. 3497549 10.2214/ajr.149.3.587

[10] Debrun GM. Angiographic workup of a carotid cavernous sinus fistula (CCF) or what information does the interventionalist need for treatment? Surg Neurol. 1995;44:75–9. 7482258 10.1016/0090-3019(95)00162-x

[11] Serbinenko FA. Balloon catheterization and occlusion of major cerebral vessels. J Neurosurg. 1974;41:125–45. 4841872 10.3171/jns.1974.41.2.0125

[12] Higashida RT, Halbach VV, Tsai FY, Norman D, Pribram HF, Mehringer CM, Hieshima GB. Interventional neurovascular treatment of traumatic carotid and vertebral artery lesions: results in 234 cases. AJR Am J Roentgenol. 1989;153:577–82. 2763958 10.2214/ajr.153.3.577

[13] Teng MM, Chang CY, Chiang JH, Lirng JF, Luo CB, Chen SS, Chang FC, Guo WY. Double-balloon technique for embolization of carotid cavernous fistulas. AJNR Am J Neuroradiol. 2000;21:1753–6. 11039361 PMC8174863

[14] Gemmete JJ, Ansari SA, Gandhi DM. Endovascular techniques for treatment of carotid-cavernous fistula. J Neuroophthalmol. 2009;29:62–71. 19458580 10.1097/WNO.0b013e3181989fc0

[15] Vollherbst DF, Cekirge HS, Saatci I, et al. First clinical multicenter experience with the new pipeline vantage flow diverter. J Neurointerv Surg. 2023;15:63–9. 10.1136/neurintsurg-2021-018480. 35172983 10.1136/neurintsurg-2021-018480

[16] Wendl CM, Henkes H, Martinez Moreno R, Ganslandt O, Bäzner H, Aguilar Pérez M. Direct carotid cavernous sinus fistulae: vessel reconstruction using flow-diverting implants. Clin Neuroradiol. 2017;27(4):493–501. 10.1007/s00062-016-0511-6. Epub 2016 Apr 29. PMID: 27129454; PMCID: PMC5719129. 27129454 10.1007/s00062-016-0511-6 PMC5719129

